# Management of a Gastrobronchial Fistula Presenting 5 Years After a One-Loop Gastric Bypass

**DOI:** 10.14309/crj.0000000000000570

**Published:** 2021-05-12

**Authors:** Toufic Saber, Saleem Abdel Backi, Charbel Aoun, Elie Ghabi, Ziad El Rassi

**Affiliations:** 1 Saint Georges Hospital University Medical Center, Beirut, Lebanon; 2 University of Balamand Faculty of Medicine and Health Sciences, Beirut, Lebanon

## Abstract

Anastomotic leaks and gastric fistulas are recognized complications after sleeve gastrectomy and Roux-en-Y gastric bypass. They are, however, almost unheard of following a one-anastomosis gastric bypass. A gastrobronchial fistula, an exceedingly rare complication after bariatric surgery, has to date never been described following a one-anastomosis gastric bypass. Furthermore, there is no consensus regarding the management of this challenging complication. In our case, we present a patient who was discovered to have a gastrobronchial fistula 5 years after a one anastomosis gastric bypass. After 2 failed attempts at endoscopic stent placement, the patient was successfully managed with a laparoscopic Roux-en-Y gastrojejunostomy over the fistula.

## INTRODUCTION

Fistula formation after bariatric surgery is becoming a more recognized phenomenon with an incidence of 8.3% following sleeve gastrectomy (SG) and Roux-en-Y gastric bypass (RYGB).^1^ Fistula formation is associated with prior gastric leak. With an incidence of 0.1%, gastric leak is rarely encountered after a one-anastomosis gastric bypass (OAGB).^2^ It is more frequently encountered after laparoscopic RYGB and SG with an incidence of 2-5.2% and 2.5%, respectively.^3^ Fistulas can be gastropleural, gastrocutaneous,^4^ gastrogastric,^5^ or, in exceedingly rare situations, gastrobronchial. Although their incidence is mainly unknown, gastrobronchial fistulas (GBFs) are more frequently described after SG rather than RYGB.^3^ A GBF occurring after an OAGB, however, has never been previously described. In this article, we present a 55-year-old man who was discovered to have a GBF 5 years after an OABG during a workup for recurrent pulmonary infections. The patient was successfully managed with laparoscopic RYGB over the fistula after 2 failed attempts at endoscopic stent placement. To our knowledge, this is the first case of a GBF occurring after an OABG.

## CASE REPORT

**Figure 1.**
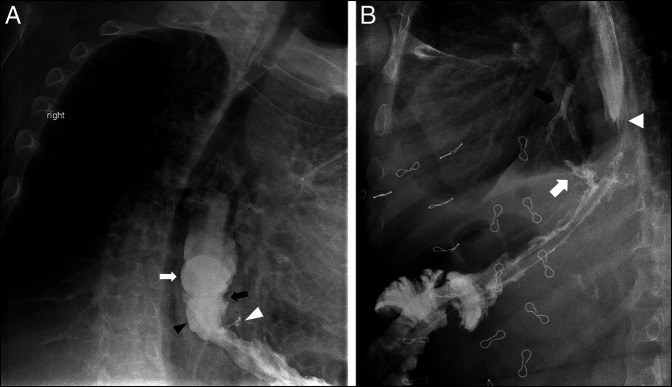
Upper gastrointestinal barium meal demonstrating (A) the distal esophagus (white arrow), the esophagogastric junction (black arrow), the gastric pouch (black arrowhead), and the fistulous tract (white arrowhead) and (B) the fistulous tract (white arrow), contrast in the left main bronchus (black arrow) and the esophagogastric junction (white arrowhead).

Our patient is a 55-year-old man who was referred to our institution for the management of a diagnosed gastrobronchial fistula. His surgical history is significant for an adjustable gastric band placed 35 years earlier followed by an OAGB performed 5 years before presentation to achieve better weight loss. The decision to perform an OAGB instead of an RYGB was based on surgeon preference. Five years after the OAGB, the patient developed a persistent cough and recurrent pneumonia. During the workup, an upper gastrointestinal (GI) barium swallow revealed evidence of a GBF involving the left main bronchus (Figure 1). Gastroscopy was performed and revealed a fistula present at 1-2 cm distal to the gastroesophageal junction. Bronchoscopy was not performed at the time. Our patient was properly diagnosed with an upper GI barium swallow, which revealed contrast filling of the left lower lobe of the lung and gastroscopy and computed tomography (CT) scan.

Endoscopic management of the fistula was attempted twice. Stent migration was encountered after both attempts. Failure of endoscopic management was attributed to the technically challenging location of the fistula. Because of technical limitations at our institution, other methods of endoscopic management were not attempted. The patient was re-evaluated and offered a laparoscopic RYGB.

**Figure 2.**
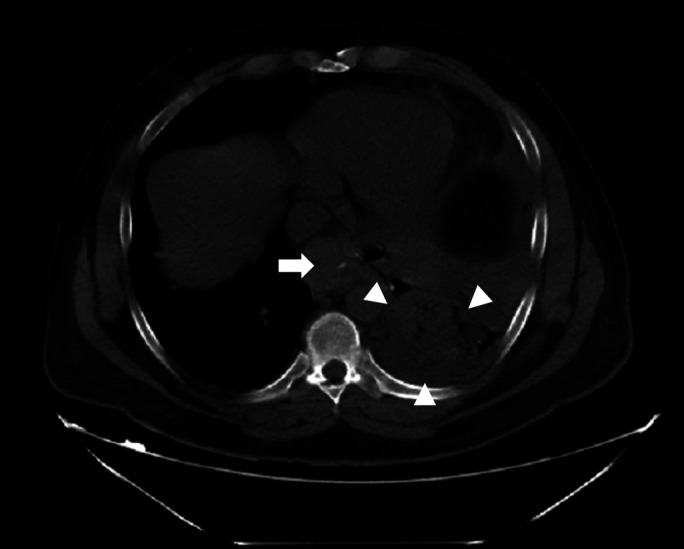
Noncontrast computed tomography scan demonstrating the gastric pouch (arrow) and the lung consolidation (arrowheads).

**Figure 3.**
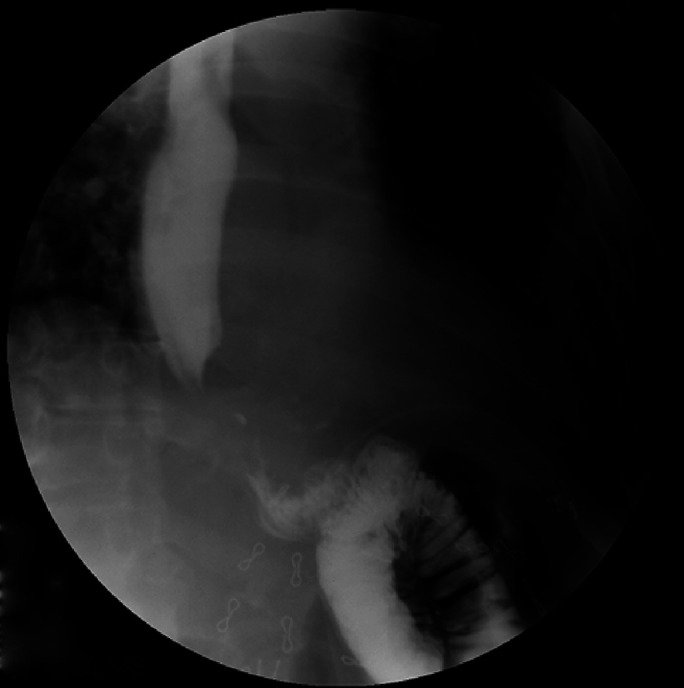
Postoperative upper gastrointestinal barium meal showing no evidence of a residual fistulous tract.

Intraoperatively, the OAGB was identified and reversed. Adhesiolysis and hydrodissection was performed, and the fistula was identified approximately 2 cm distal to the gastroesophageal junction. The fistula was ablated, and a 1 × 1-cm opening was identified in the gastric pouch (Figure 2). The edges of the opening were refreshed, and then, a fistula-jejunostomy was performed over the opening. An upper GI barium swallow performed on postoperative day 2 demonstrated complete resolution of the fistula (Figure 3).

The patient was started on a triphasic diet for dietary progression. A triphasic diet begins with a 10-day phase of a complete liquid diet, followed by a phase of a semisolid diet and, finally, a phase of a regular solid diet. Double-dose proton-pump inhibitor therapy was also initiated, and the patient was discharged after an uncomplicated hospitalization. On the 1-month follow-up visit, the patient reported tolerating a regular solid diet and denied recurrence of cough or pulmonary infections.

## DISCUSSION

**Figure 4.**
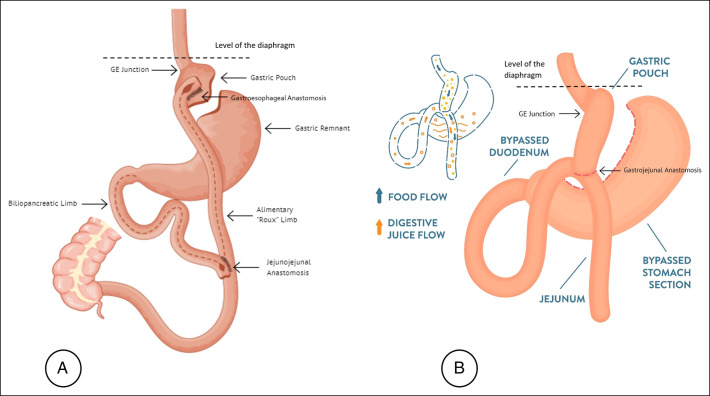
Comparison between the (A) conventional Roux-en-Y gastric bypass and the (B) anastomosis gastric bypass. GE, gastroesophageal.

The increase in performed bariatric surgeries parallels the increasing global burden of morbid obesity.^5^ Surgery remains the mainstay treatment to achieve weight loss and reduce the risks of diabetes, hypertension, and other obesity-related comorbidities.^6^ The OABG, as first reported by Rutledge, did not garner as strong a following as SG or RYGB despite having comparable expected weight loss at 1 and 2 years and a comparable rate of complications at 5.2%.^5^ In a recent study by Taha et al,^2^ 3.3% of 1,520 patients who underwent OABG developed complications, with abdominal bleeding being the most common early complication (1.7%) and iron deficiency anemia the most common late complication (3.1%). An anastomotic leak was observed in 1 patient requiring conversion to RYGB.^2^ Gastrobronchial fistulas are rarely encountered after bariatric surgery (Figure 4). The reported cases occurred after an SG more so than an RYGB^2,3,6–10^ and even gastric banding,^11^ but never after an OABG. A gastropleural fistula, however, has been described after an OABG,^12^ making it the only fistula ever encountered. In terms of surgical technique, a gastric pouch is created similarly to that in an RYGB. Thus, similar conditions should lead to leaks and fistula formation.^13^ Fistulas are rarely observed because OAGB has not been widely adopted until recently. Therefore, as it becomes more routinely performed, fistulas may be encountered more often.

Interestingly, the gastric band may have predisposed the patient to a gastric leak, possibly secondary to impaired circulation along the staple line of the gastric pouch where the band was previously in place. In general, fistulas form following a leak that leads to a subphrenic abscess.^3^ Given its proximity to the respiratory tract,^3^ the infection creates a fistulous tract that occasionally forms a gastrophrenic fistula and rarely a GBF.^14^ In a series by Bruzzi et al,^14^ a GBF involved the left lower lobe of the lung in 9 of 11 patients, whereas 2 of 11 had a transhiatal course. In our case, a transhiatal course was observed.

In the review by Silva et al,^3^ a GBF was diagnosed between 1 and 30 months, with a mean of 7.2 months, after bariatric surgery. Infectious or respiratory symptoms such as fever, productive cough, vomiting, and recurrent pneumonia were frequently reported.^3^

Workup of a GBF generally involves a thoracic x-ray, an upper GI series, a CT scan, or a gastroscopy.^3^ When pulmonary involvement is suspected, it is preferable to use a barium meal rather than Gastrografin during the upper GI series because it is associated with pulmonary complications such as edema or respiratory failure.^15^ A barium meal is, therefore, more suitable. Bronchoscopy is not routinely used. It was attempted, but a fistula orifice could not be identified.^3^

Given the rarity of this condition, management is highly controversial. Endoscopic management with stent placement, fibrin glue, or stricturotomy and septoplasty could be attempted by an experienced endoscopist for a suitable fistulous tract.^3^ Surgery is also a valid option with multiple approaches and corrections described, of which conversion to RYGB has been frequently and consistently reported.^3^ Therefore, surgical management is a successful strategy should an endoscopic approach fail or prove technically challenging.

## DISCLOSURES

Author contributions: T. Saber wrote the manuscript and reviewed the literature. SA Backi and C. Aoun wrote the manuscript and provided the images. E. Ghabi edited the manuscript and reviewed the literature. Z. El Rassi revised the manuscript for intellectual content, approved the final manuscript, and is the article guarantor.

Financial disclosure: None to report.

Informed consent was obtained for this case report.
